# Research Note: Probiotic, *Bacillus subtilis,* alleviates neuroinflammation in the hippocampus via the gut microbiota-brain axis in heat-stressed chickens

**DOI:** 10.1016/j.psj.2023.102635

**Published:** 2023-03-09

**Authors:** Yuechi Fu, Jiaying Hu, Heng-wei Cheng

**Affiliations:** ⁎ Department of Animal Sciences, Purdue University, West Lafayette, IN 47907, USA; † USDA-Agricultural Research Service, Livestock Behavior Research Unit, West Lafayette, IN 47907, USA

**Keywords:** chicken, heat stress, innate immunity, neuroinflammation, probiotic

## Abstract

High ambient temperature (heat stress, **HS**) is one of the critical environmental factors causing gut microbiota dysbiosis and increasing gut permeability, consequently inciting neuroinflammation in humans and various animals including chickens. The aim of this study was to examine if a probiotic, *Bacillus subtilis*, can reduce neuroinflammation in heat-stressed broiler chickens. Two hundred and forty 1-d-old broiler chicks were randomly assigned to 48 pens among 4 treatments in 2 identical, thermal-controlled rooms (*n* = 12): Thermoneutral (**TN**)-regular diet (**RD**), TN-**PD** (the regular diet mixed with a probiotic at 250 ppm), HS-RD, and HS-PD. The probiotic diet was fed from d 1, and HS at 32°C for 10-h daily was applied from d 15 for a 43-day trial. Results showed that compared to the TN broilers, the HS broilers had higher hippocampal interleukin (**IL**)-6, toll-like receptor (**TLR**)4, and heat shock protein (**HSP**)70 at both mRNA and protein levels regardless of dietary treatment (*P* < 0.05). In addition, the HS-PD broilers had higher levels of hippocampal IL-8 (*P* < 0.05) than the TN-PD broilers. Within the HS groups, compared to the HS-RD broilers, the HS-PD broilers had lower levels of IL-6, IL-8, HSP70, and TLR4 (*P* < 0.05) in the hippocampus. Within the TN groups, the TN-PD broilers had lower IL-8 at both mRNA expressions and protein levels (*P* < 0.05) but higher TLR4 protein levels (*P* < 0.05) in the hippocampus as compared to the TN-RD broilers. These results indicate that dietary supplementation of the *Bacillus subtilis*-based probiotic may reduce HS-induced brain inflammatory reactions in broilers via the gut-brain-immune axis. These results indicate the potential use of probiotics as a management strategy for reducing the impact of HS on poultry production.

## INTRODUCTION

Heat stress (**HS**) is a critical health risk in humans and animals, including chickens. Chickens are particularly sensitive to heat due to the breeding selection (meat or eggs), leading to intense metabolic heat production and reduced capability of thermoregulation. The chicken health and welfare issues have become much worse with recent climate change increasing heat waves. In the summer of 2022, fatal heat with the worst heat waves swept across the world and frequently broke a daily record (Rosenthal and Patel, 2022). In addition, the ability of chickens to lose body heat is limited by feathering and the absence of sweat glands. Heat stress negatively impacts feed intake, disrupts energy metabolism, causes oxidative stress, immunosuppression, and homeostatic imbalance, consequently, reduces reproduction and growth performance, resulting in great economic loss, especially in the tropical and subtropic regions (Jiang et al., 2021).

Heat stress as a major environmental stressor causes gut microbiota dysbiosis, releasing proinflammatory factors such as interleukin (**IL**)-1, IL-6, and tumor necrosis factor (**TNF**)-alpha, by which it affects brain function via multiple pathways of the microbiota-gut-brain (**MGB**) axis. The hippocampus, as a subcortical region, is highly sensitive to chronic stress, including HS, and stress-induced overexpression of those proinflammatory cytokines in the hippocampus has been used as biomarkers of neuroinflammation in humans (Johnson et al., 2019). Several probiotics such as *Lactobacillus plantarum* DP189 and *Bifidobacterium breve* MCC1274 have been used as novel biotherapeutic agents for reducing gut dysbiosis effects on neuroinflammation-associated brain damage and neuropsychiatric disorders in humans (Queiroz et al., 2022). In chickens, *Bacillus subtilis* has been used as dietary supplementation or an alternative to antibiotics in poultry under various conditions including HS (Wang et al., 2018). In our early studies, we have found that dietary supplementation of *Bacillus subtilis* improves performance, meat quality, skeletal health, behavior, and systemic immunity in broilers exposed to heat at 32°C for 10 h daily from d 15 to d 42 (Wang et al., 2018; Jiang et al., 2021). Following HS, the probiotic-fed broilers have lower heterophil-to-lymphocyte ratios (a stress indicator), lower concentrations of hepatic IL-6, and spend less time exhibiting HS-associated behaviors such as panting and wing spreading as compared to the control broilers (Wang et al., 2018). These results indicate that broilers fed with *Bacillus subtilis* can cope with HS more effectively by ameliorating heat-induced behavioral and inflammatory reactions through regulation of the microbiota-gut-immune axis. However, the effects of *Bacillus subtilis*-based probiotics on the immune response of brain in HS broilers have not been previously investigated. Therefore, the objective of this study was to examine if dietary *Bacillus subtilis* supplementation ameliorates inflammatory reactions in the hippocampus of broilers exposed to HS.

## MATERIALS AND METHODS

The project was approved by the Animal Care and Use Committee of Purdue University, and the animals were housed at the Animal Research and Education Center of Purdue University in accordance with the guidelines of the Federation of Animal Science Societies (McGlone, 2010).

### Birds and Experimental Design

Two hundred and forty 1-d-old broiler male chicks (Ross 708 strain) were used in this study. The chicks were weighed in 5 bird-group and assigned to 48-floor pens (152 cm × 81 cm each pen) with equal weight distribution among the pens. The pens were evenly assigned into 4 treatments in each of 2 identical, temperature-controlled rooms (*n* = 12): 1) thermoneutral (**TN**) condition—a regular diet (**RD**); 2) TN-**PD** (the regular diet mixed with a probiotic, 250 ppm Sproulin); 3) HS-RD; and 4) HS-PD. The concentration of *Bacillus subtilis*-based probiotic (1 × 10^6^ CFU/g feed) was recommended by the company (Novus International, Inc., St. Louis, MO) and has been tested in our pilot studies. The probiotic dietary treatment was started from d 1. The nutritional levels of the starter (d 1–14), grower (d 15–28), and finisher (d 29–43) met or exceeded the dietary recommendations by Aviagen (Wang et al., 2018). The temperature of one room was reduced 0.5°C per d from 34°C on d 1 until 21°C ± 1°C, while in another room, heat stimulation, 32°C ± 1°C for 10 h (07:00–17:00) daily, was applied at d 15 until the end of this study. Throughout the study, water and feed were provided ad libitum and the birds were raised under the conditions reported previously (Wang et al., 2018).

### Sample Collection

At d 43 during the HS period, each sampled broiler (1 bird per pen, *n* = 12 per treatment) was sedated by injection of sodium phenobarbital (30 mg/kg BW, iv; Sigma-Aldrich, St. Louis, MO) via the brachial vine within 2 min of removed from home pen. Following euthanasia, the hippocampus was dissected from each sampled bird within 3 min based on the brain anatomical land markers described previously (Puelles et al., 2007). All samples were snap-frozen and then stored at −80°C until analysis.

### RT-qPCR Analysis

The total RNA of each hippocampal sample was extracted by using RNeasy Mini Kits (Catalog #: 74804, Qiagen Inc., Valencia, CA) following the instructions provided by the company, then the purity and quantitation were examined using the GeneQuantTM 100 Spectrophotometer (GE Healthcare, Chicago, IL) as described previously (Wang et al., 2018). cDNA was synthesized by using the Maxima First Strand cDNA Synthesis Kits by following the manufacturer's protocol (Applied Biosystems, Foster City, CA). Chicken IL-6, IL-8, heat shock protein 70 (**HSP70**), and toll-like receptor 4 (**TLR4**) primers were designed and synthesized by the company (Applied Biosystems, Foster City, CA) and RT-PCR was performed by using the TaqMan Gene Expression Assays (Applied Biosystems, Foster City, CA). Briefly, the cycling conditions were 50°C for 2 min and 95°C for 10 min of the holding stage, followed by 40 cycles of 95°C for 15 s, then 60°C for 1 min. The glyceraldehyde 3-phosphate dehydrogenase (**GAPDH**) gene was used as the reference gene. Standards and samples were measured in duplicates with a CV of less than 2.0%. Data of each gene were expressed as relative quantification (**RQ**) and compared the Ct value of the targeted gene to the reference control gene using the formula: 2^ΔΔCT^ and a coefficient of variation less than 2.0%.

### ELISA Analysis

The effects of *Bacillus subtilis* on the concentrations of IL-6 (NeoScientific, Cambridge, MA; Catalog No. CKI0013), IL-8 (CKI00063), TLR4 (CKI0076), and HSP70 (CKH0029) in the hippocampus were measured using commercially available chicken ELISA kits by following the relative company's protocols. Samples were analyzed in duplicates with CV ≤ 15%. Concentrations of targeted proteins were reported as pg/mL or ng/mg (Wang et al., 2018).

### Statistical Analysis

Data were analyzed using PROC MIXED procedure of SAS 9.2 (SAS Institute Inc., Cary, NC). The pen was the experimental unit. Data were reported as least square means ± SEM. The SLICE option was used to examine the effect of one independent variable within a level of the second independent variable. The Benjamini-Hochberg method was used to control the false discovery rate due to multiple comparisons and the Tukey-Kramer test was used to partition any significant differences among the least square means due to treatment effects. Statistical significance was set at *P* ≤ 0.05, and the trend was taken at 0.05 < *P* ≤ 0.10.

## RESULTS AND DISCUSSION

The current data revealed that dietary *Bacillus subtilis*-based probiotic improved brain immunity of broilers, especially under HS, by reducing proinflammatory cytokines including IL-6 and IL-8 at both transcriptional and protein levels as well as reduced HS-upregulated HSP70 and TLR4 expressions. The results are consistent with our previous findings that *Bacillus subtilis* supplementation decreases heat-related behaviors such as panting and wing spreading; and improves systemic innate immunity by reducing hepatic levels of IL-6 but increasing hepatic levels of IL-10, an anti-inflammatory cytokine, while reduces HSP70 in broilers exposed to HS (Wang et al., 2018). The current and previous results indicate that the *Bacillus subtilis*-based probiotic may alleviate HS-induced neuroinflammation via regulation of the MGB axis.

**Figure 1:**
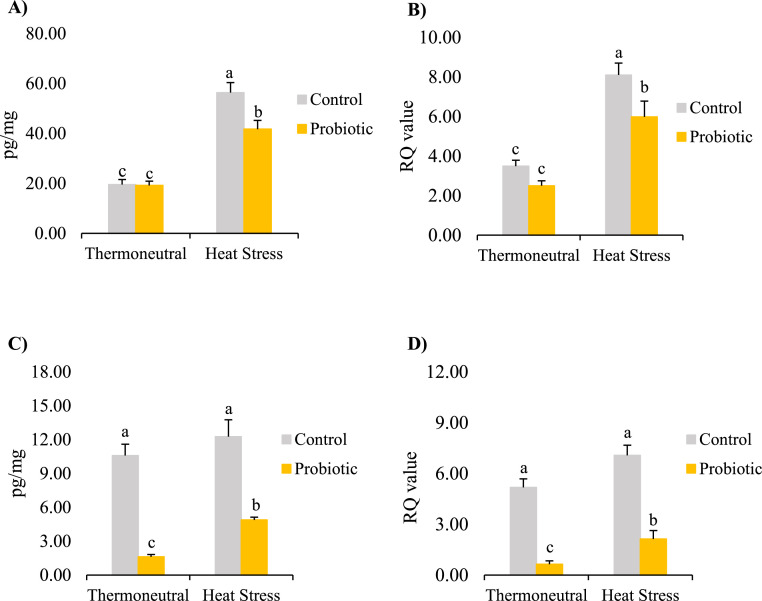
The effects of temperature and *Bacillus subtilis*-based probiotic on hippocampal (A) IL-6 protein concentrations, (B) IL-6 mRNA expression, (C) IL-8 protein concentrations, and (D) IL-8 mRNA expressions in broilers at 43 d of age. Values are least square means ± SEM (*n* = 12/treatment). a–c: *P* < 0.05.

Cytokines of the innate immune system sense various stimulations to regulate inflammatory pathways promoting physiological homeostasis, while under chronic stimulation conditions (allostatic overload), a hyperinflammation-shaped immune response results in cytokine storm leading to immunopathogenic events causing tissue injury. IL-6 is a vital proinflammatory cytokine that mediates innate immune reactions in promoting inflammation. Overexpression of IL-6 has been used as a biological marker driving inflammation in responding to a wide range of pathophysiological and immune disorders. Targeting IL-6 signaling has become a biotherapeutic strategy for neuroinflammation-promoted neuronal disorders. In the current study, hippocampal IL-6 protein levels and mRNA expressions were increased in the broilers exposed to HS regardless of dietary treatment (*P* < 0.05, Figure 1A and B). Similarly, a proinflammatory response in chicken cerebellum and cerebrum with increased IL-6 levels can be induced by various stressors such as high levels of ammonia (Wang et al., 2022). IL-8 (or chemokine C-X-C motif ligand, CXCL8) is another critical proinflammatory cytokine, which can be rapidly released from intestinal enterochromaffin cells, up to 10- to 100-folds, in response to various stimuli such as antigens of bacterial, viral, or cellular stress products (Klapproth and Sasaki, 2010). In the current study, however, hippocampal IL-8 concentrations and mRNA expressions in broilers was not affected by HS (*P* > 0.05, Figure 1C and D). It may indicate that HS-induced inflammatory reaction in broiler hippocampal was mainly via IL-6 instead of the IL-8 pathway. The differential roles of cytokines, IL-6 and IL-8 in response to traumatic brain injury have been reported previously, and IL-6 is associated with adverse outcomes of injury (Abboud et al., 2016). The current results further support the hypothesis that although the secretion profiles and signaling cascades of cytokines often overlap in response to stimuli, they are structurally and functionally distinct from one another, which has been revealed in cancers and autoimmune diseases (Patra et al., 2020).

**Figure 2:**
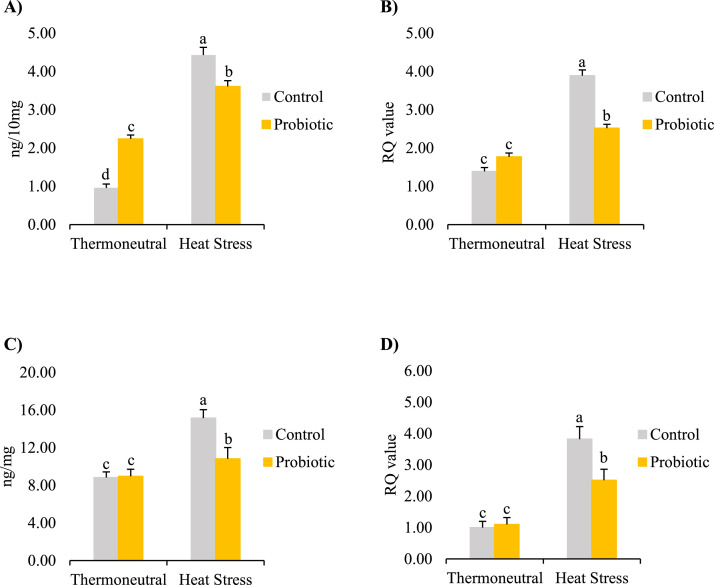
The effects of temperature and *Bacillus subtilis*-based probiotic on hippocampal (A) TLR4 protein concentrations, (B) TLR4 mRNA expressions, (C) HSP70 protein concentrations, and (D) HSP70 mRNA expressions in broilers at 43 d of age. Values are least square means ± SEM (*n* = 12/treatment). a–c: *P* < 0.05.

TLR4 is the most important member of the pattern recognition receptor family, playing a fundamental role in recognizing danger-associated signals from damaged cells and pathogens, and rapidly responding with activated innate immune reactions. Especially, TLR4, as a microbe-sensing receptor, recognizes pathogen-derived molecules such as lipopolysaccharides (**LPS**) to promote an immune response against infection and inflammation by bridging the innate and adaptive immune systems (Candelli et al., 2021). The LPS-TLR4 route has been used as a reliable molecular biomarker for endotoxicity and related brain damage. In the current study, overproduced TLR4 was found in the hippocampus of HS broilers regardless of dietary treatments (*P* < 0.05, Figure 2A and B). Similarly, overregulation of TRL4 in the hippocampus has been found in heat stress-induced neuroinflammation in mice (Huang et al., 2022).

HSP70, a member of stress-inducible proteins (HSPs, previously called stress proteins), acts as a molecular chaperone to protect cells from reactive oxygen species (**ROS**) caused by intracellular protein denaturation (misfolding or aggregation). Notably, HSP70 as an immunomodulant is also implicated in both pro- and anti-inflammatory responses. Studies have shown that HSP70 is inducible in both broiler chickens and laying hens when exposed to HS; and the HSP70 expression is positively correlated with the degree of heat stimulation (its duration and intensity) and related cell damage (Wang et al., 2018). Under high temperatures, the expression of HSP70 in a chicken, similar to its function in mammals, is responded to overly produced ROS in multiple organs including the brain (Jiang et al., 2020). In the current study, upregulated HSP70 at both mRNA and protein levels were found in the hippocampus of HS broilers (*P* < 0.05, Figure 2C and D). Similarly, HS-stimulated HSP70 expression is increased for neuroprotection in humans and experimental animals suffering from temperature shock, brain injury, or various inflammatory disorders (Lee et al., 2006). The current and previous results indicate that HPS70 is involved in HS-induced neuroinflammation, which may be related to the inputted signal from the interrupted gut microenvironment to the brain via the MGB axis. Although HS effects on gut microbiota has not been examined in the current study, 16S rRNA gene sequencing in the previous studies has revealed HS altered gut microbial community in various animals including chickens (Jin et al., 2022).

Probiotics offer potential health-beneficial effects in immunomodulation, metabolic homeostasis, and neuroendocrine regulation. Among the bacterial species, probiotic *Bacillus subtilis* has been widely used as an alternative to antibiotics in poultry production (Jiang et al., 2021). *Bacillus subtilis* inhibits systemic inflammation in broiler chickens via preventing pathogens from binding to intestinal epithelia, altering the expression of cytokines in the intestinal intraepithelial lymphocytes, and releasing antimicrobial chemicals and neurotransmitters (Jiang et al., 2021). In our previous study, *Bacillus subtilis* reduced heat stress-induced hepatic IL-6 expression in (Wang et al., 2018). In the current study, *Bacillus subtilis* decreased synthesis and secretion of cytokines, such as IL-6 and IL-8, in the hippocampus of broilers exposed to HS. Compared to the HS-RD birds, the HS-PD birds had lower protein concentrations and mRNA expression of IL-6 (Figure 1A and B, *P* < 0.05). Compared to each relative control, both the TN-PD and HS-PD birds had lower levels of IL-8 under both TN and HS conditions (Figure 1C and D, *P* < 0.05). Similar to our findings, *Bacillus subtilis* protects mouse hippocampal cells from neurotoxin damage by releasing surfactin to inhibit production of ROS and suppress the expressions of IL-1β, IL-6, and TNF-α, and surfactin reduces the amyloid β-induced inflammatory reactions in microglial cells which serve as neuroimmune modulators in the central nervous system to monitor various stimulations (de Moura et al., 2022).

The current results revealed that *Bacillus subtilis*-based probiotic alleviates HS-induced brain inflammation via regulation of TLR and HSP pathways. Compared to the HS-RD group, the probiotic alleviated the HS-induced elevated TLR4 and HSP70 at both mRNA and protein levels (Figure 2, *P* < 0.05) in the hippocampus of the HS-PD broilers. Within the TN group, higher protein levels of hippocampal TLR4 in the TN-PD broilers (compared to the TN-RD control) may indicate that *Bacillus subtilis* can stimulate TLR4 synthesis and release at a level range which can be further stimulated under extreme conditions such as HS. Under HS, TLR4 at protein concretions and mRNA expressions were increased in both HS-RD and HS-PD birds, while the reactions were much lower in HS-PD birds. The protective effects of activated TLR4 signaling pathway, such as LPS-TLR4, and its neuroimmune regulation effects have been widely studied in humans and various animals. It has been reported that an enhanced TLR4 reaction inhibits the proinflammatory IL-6 trans-signaling via the STAT3 pathway in an LPS/TRL4-mediated septic shock mouse model (Greenhill et al., 2011). Similarly, it has been observed that the chickens challenged with pathogens have higher TLR4 mRNA levels in the spleen and cecum (Yang et al., 2022). In the current study, the pathogens or LPS challenge was not used based on the study design, but our results support that there is a HSP70-TLR4 signaling pathway in preventing oxidant-induced neuroinflammation (Candelli et al., 2021).

The current results revealed that the *Bacillus subtilis*-based probiotic activated the HSP70-TLR4 signaling pathway, displaying an important cytoprotective effect in the hippocampus under HS conditions. Although the mechanism of its regulation has not been examined in this study, it could be similar to the ones reported in mammals: HSP70 functions as an endogenous stimulator to induce TLR4 activation via innate and adaptive immune responses, consequently, protects cells from HS-induced neuroinflammation (Candelli et al., 2021). In addition, probiotics may reduce the translocation of HS-associated damage signals and related glial activation via the MGB axis. Previous studies have reported that the level of serotonin in the raphe nuclei is increased whereas the concentrations of norepinephrine and dopamine are reduced in the hypothalamus of broilers fed with the probiotic (Yan et al., 2018). The signaling from the stress-disrupted gut homeostasis (leaky gut) can be transferred to the brain via multiple pathways influencing brain function. Previous studies have reported that *Bacillus subtilis* modulates gut microbiota composition in chickens (Zou et al., 2022). Taken together, our data suggest that the dietary probiotic reduces neuroinflammation in broilers through regulating the MGB axis. Further studies will be conducted to investigate the signaling pathways of the MGB axis in responding to the effects of *Bacillus subtilis* on HS broilers.
